# Arthroscopic repair is an effective treatment for dynamic medial ankle instability secondary to posttraumatic and partial injury of the deltoid ligament deep fascicle

**DOI:** 10.1002/ksa.12197

**Published:** 2024-05-01

**Authors:** Jordi Vega, Francesc Malagelada, Matteo Guelfi, Miki Dalmau‐Pastor

**Affiliations:** ^1^ Foot and Ankle Unit iMove Traumatology Barcelona Spain; ^2^ Foot and Ankle Unit Olympia Madrid Spain; ^3^ Laboratory of Arthroscopic and Surgical Anatomy, Department of Pathology and Experimental Therapeutics (Human Anatomy Unit) University of Barcelona Barcelona Spain; ^4^ MIFAS by GRECMIP Merignac France; ^5^ Department of Trauma and Orthopedic Surgery, Royal London Hospital Barts Health NHS Trust London UK; ^6^ Casa di Cura Villa Montallegro Genova Italy; ^7^ Department of Orthopaedic Surgery “Gruppo Policlinico di Monza” Clinica Salus Alessandria Italy

**Keywords:** all‐inside repair, ankle, arthroscopy, instability, medial ankle instability

## Abstract

**Purpose:**

When the intermediate or collicular fascicle of the medial collateral ligament (MCL) is injured, the diagnosis of posttraumatic medial ankle instability (MAI) is supported. The aim of this study was to describe an arthroscopic all‐inside MCL repair after posttraumatic MAI secondary to an isolated injury of the MCL deep fascicle with a knotless suture anchor technique.

**Methods:**

Seven patients (seven men, median age: 23 [19–28] years) with posttraumatic MAI were treated by arthroscopic means after failing nonoperative management. The median follow‐up was 34 (13–75) months. The MCL was repaired with an arthroscopic all‐inside technique.

**Results:**

A tear affecting the deep and intermediate or collicular fascicle of the MCL was observed in all cases. In addition, five patients were diagnosed with an isolated fibular anterior talofibular ligament (ATFL) detachment, and in two patients, both the ATFL and calcaneofibular ligament were involved. All patients reported subjective improvement after the arthroscopic ligament repair. The median American Orthopedic Foot and Ankle Society score increased from 68 (range: 64–70) preoperatively to 100 (range: 90–100) at final follow‐up.

**Conclusion:**

Posttraumatic MAI can be successfully treated by an arthroscopic all‐inside repair of the MCL. The presence of an MCL tear affecting the tibiotalar ligament fibres attached to the area of the anterior colliculus should be considered a sign of posttraumatic MAI. This partial deltoid injury at the level of the intermediate or collicular fascicle will conduct to a dynamic MAI.

**Level of Evidence:**

Level IV.

**Abbreviations**
ATFL: anterior talofibular ligament
CFL: calcaneofibular ligament
MAI: medial ankle instability
MCL: medial collateral ligament
MRI: magnetic resonance imaging

## INTRODUCTION

The medial collateral ligament (MCL) of the ankle or deltoid ligament is the primary stabilizer against valgus forces to the ankle, and it is subject to acute or chronic injuries. The mechanism of injury leading to acute MCL damage involves a force in pronation and external rotation or, less commonly, in supination and external rotation [20]. Depending on the amount of force imparted during the injury, it may lead to a malleolar fracture, syndesmotic disruption, MCL injury or a combination of those. Isolated posttraumatic MCL injuries without ankle fractures are a rare occurrence but a known entity [7, 26].

Acute surgical repair is not commonly proposed after acute MCL complex injuries, and conservative therapy is favoured [21, 28]. However, a number of patients undergoing conservative treatment may develop medial ankle instability (MAI) [13, 19]. MAI is defined as an abnormal feeling of giving way medially when walking or during sports, and typically an asymmetric hindfoot valgus can be observed on weight bearing.

Recently, some authors have developed arthroscopic techniques to treat MCL ligament injuries with excellent results [1, 18, 29]. However, to date, no similar arthroscopic techniques have been published to deal with posttraumatic MAI after a short period of immobilization and rehabilitation.

The aim of this study was to describe an arthroscopic all‐inside MCL repair after posttraumatic MAI secondary to an isolated injury of the MCL deep fascicle with a knotless suture anchor technique. The results in a series of seven patients are presented. The described technique represents the first arthroscopic all‐inside MCL repair procedure in cases of posttraumatic MAI performed in the acute/subacute phase (within 6 months from the index injury). In addition, the arthroscopic description of MCL deep fascicle injury in a posttraumatic early MAI setting was presented.

It was hypothesized that proximal or tibial detachment of the tibiotalar fascicle (deltoid ligament deep fascicle) would be a common feature in patients with symptoms of posttraumatic MAI and that an arthroscopic repair of this fascicle injury would yield excellent results.

## MATERIALS AND METHODS

Descriptive results were presented as median and range.

Between 2013 and 2020 (a 7‐year period), 529 patients were arthroscopically treated for ankle instability by the senior author. From this group, seven patients were clinically diagnosed with acute posttraumatic MAI (seven males, median age 23; range: 19–28 years). The left ankle was affected in 4 of the 7 cases. Five patients were professional football players and two were professional basketball players.

Patients included in the study had sustained one ankle eversion sprain within 6 months from surgery. They reported medial swelling and bruising after the acute injury, and tenderness was localized over the medial aspect of the ankle in the acute phase. Patients completed 2 weeks of immobilization and a minimum of 3 months of protocolized physiotherapy. No evident hindfoot pronation was observed in any case during clinical examination. Patients reported failed conservative treatment with pain or discomfort medially, as well as a subjective feeling of MAI and/or feeling of collapse of the medial arch during sports activity, especially when intense or stressful. The MAI or collapse feeling was not obtained after repetitive single leg heel raises but with running on the treadmill for more than 10–15 min.

In addition to the eversion ankle sprain, all patients reported a history of repetitive inversion sprains affecting the same ankle. Those inversion ankle sprains were successfully treated through a conservative protocol. However, six patients reported lateral ankle discomfort; four of them reported lateral ankle complaints previous to the ankle eversion trauma. A positive anterior drawer test was observed in all patients indicating lateral ankle instability. No patients reported any discomfort around the syndesmotic area. No symptoms were reported affecting the peroneal or Achilles tendons. Two patients reported complaints in the submalleolar area of the posterior tibialis tendon; however, no deficit in the power of the posterior tibialis tendon was observed.

Functional outcomes using the American Orthopedic Foot and Ankle Society (AOFAS) hindfoot score were assessed preoperatively and at the latest follow‐up (minimum of 1 year after the procedure).

No patients had sustained any foot or ankle fractures or had undergone any previous foot or ankle surgery.

**Figure 1:**
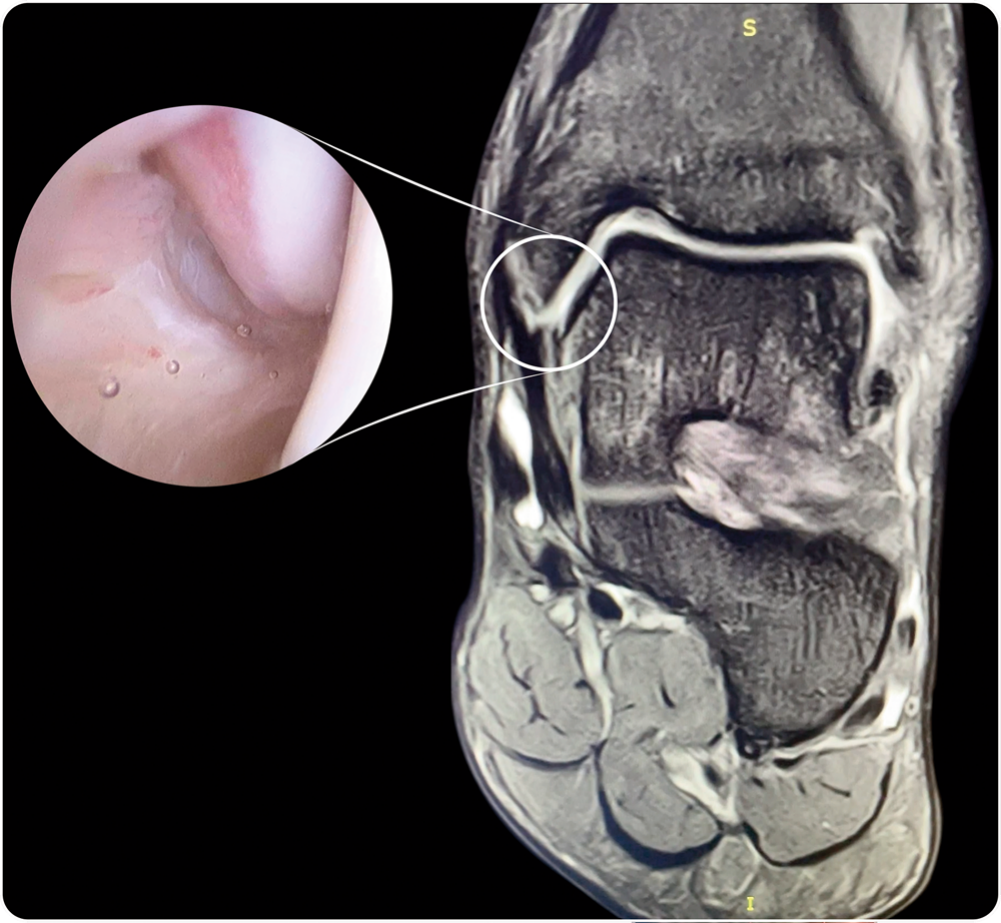
Magnetic resonance imaging showing proximal detachment of the deep fascicle at the level of the anterior colliculus. Arthroscopic view of the deltoid injury.

Plain radiographs showed an os trigonum in four of the cases and an anterior tibial osteophyte in two patients. Preoperative magnetic resonance imaging (MRI) of the ankle was obtained in all cases. MRI showed chronic anterior talofibular ligament (ATFL) injury in all cases. Although MCL injury was observed in all patients, in three cases, it showed an evident MCL detachment of the deep fascicle at the level of the medial malleolus tip or anterior colliculus (Figure 1). Superficial chondral injury at the medial talar dome was observed in two cases. No abnormalities or just synovial fluid within the tendon sheath were observed at the level of the posterior tibialis tendon. No peroneal tendons or Achilles tendon alterations were observed.

### Operative technique

Instruments used to arthroscopically repair the MCL included a 4.0 mm 30° arthroscope, an arthroscopic 3.5 mm motorized shaver and burr, and standard arthroscopic instruments. A suture passer (MiniScorpion; Arthrex), 0 nonabsorbable sutures (Fiberwire; Arthrex) and knotless suture anchors (Pushlock 2.9 mm × 15 mm; Arthrex) were also used for ligamentous repair.

Under spinal anaesthesia, the patient was positioned supine with the affected leg on an elevated thigh support placed under the knee.

An ankle dorsiflexion arthroscopic technique without distraction was performed. Anteromedial and anterolateral ankle portals were established.

Routine synovectomy or excision of any existing adhesions was carried out with a shaver. Gutters were examined, and ligaments were probed in order to assess their anatomical characteristics.

**Figure 2:**
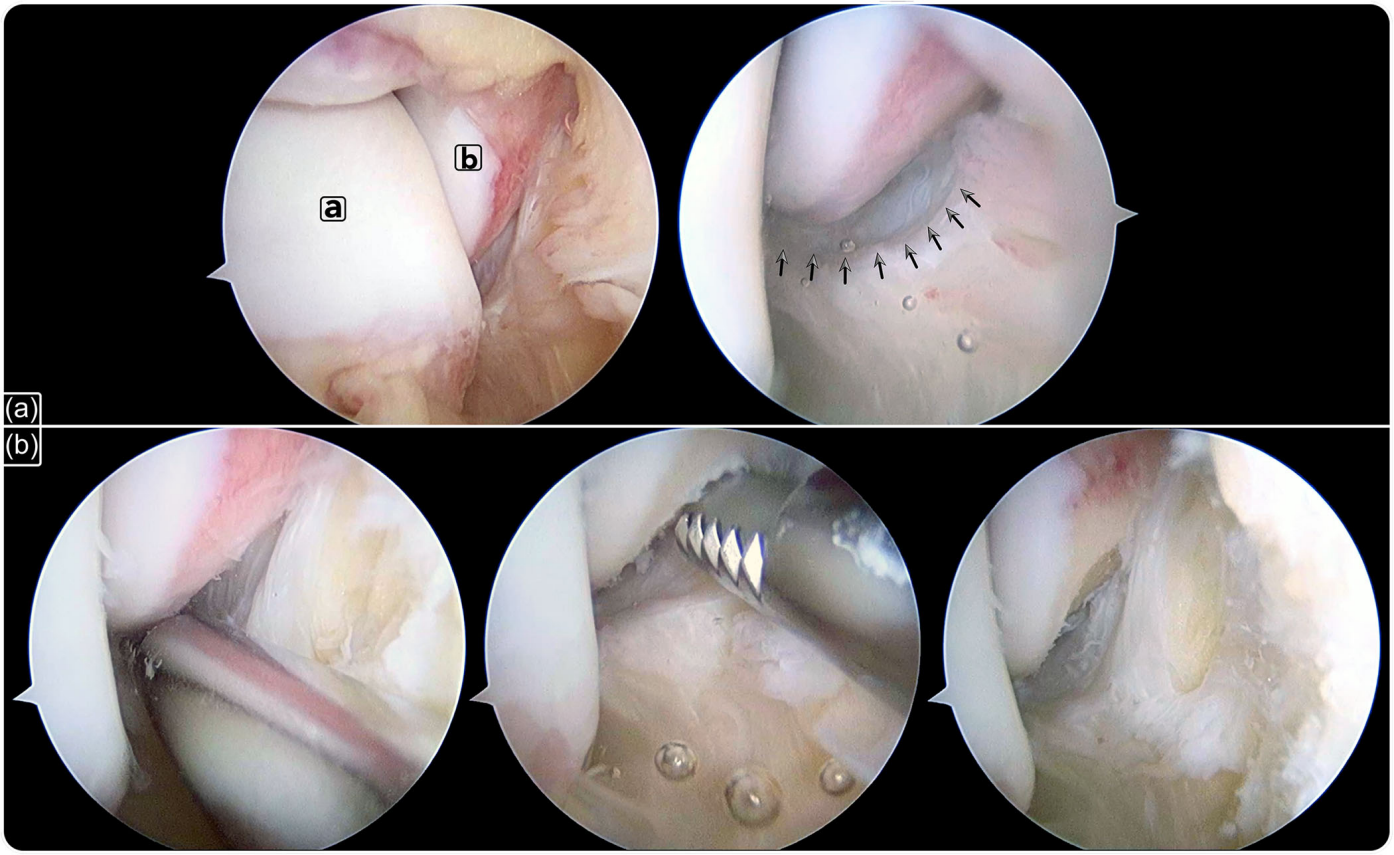
Arthroscopic view of the medial gutter in a right ankle. Scope introduced through the anterolateral portal and directed to the medial gutter. (a) Deltoid injury indicated with arrows: a. Talar dome. b. Medial malleolus. (b) Ligament footprint is debrided with a shaver introduced through the anteromedial portal.

While maintaining ankle dorsiflexion, the tibiotalar fibres are observed at the floor of the medial gutter, resembling a hammock from the medial side of the talus (just inferior to its medial articular surface) and inserts onto the medial and distal aspects of the medial malleolus. The anterior colliculus is observed as the tip of the medial malleolus. The anterior fibres of the tibiotalar fascicle are attached to the anterior aspect of the medial malleolus anterior colliculus. The posterior fibres of the tibiotalar fascicle are partially visible with the arthroscope introduced through the anterolateral portal and directed to the medial recess. Bearing in mind that the tibiotalar fascicle is the deltoid deep fascicle, its injury will modify the normal arthroscopic vision of the medial gutter featuring the presence of a ligament stump occupying the gutter when the injury is complete or resembling a 'wave sign' when partial (Figure 2a).

**Figure 3:**
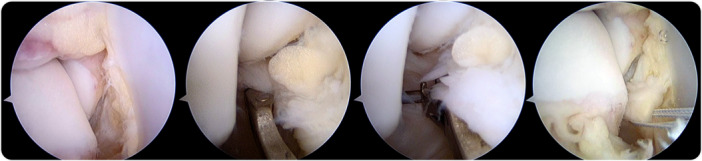
Arthroscopic view of the medial gutter in a right ankle. Scope introduced through the anterolateral portal and directed to the medial gutter. Automatic suture passer clamp introduced through the anteromedial portal. The ligament is penetrated with an automatic suture passer clamp. A double suture is used to penetrate the ligament. Next, one of the ends of the suture is introduced into the suture loop, and by pulling the ends, the loop is introduced into the joint and the ligaments are grasped by the suture.

After completing the arthroscopic examination, the tibiotalar fascicle was repaired with the arthroscope introduced through the anterolateral portal and directed towards the medial gutter. The footprint for the attachment of the tibiotalar fascicle on the medial malleolus was debrided with a shaver (Figure 2b). An automatic suture passer clamp (MiniScorpion; Arthrex) was introduced through the anteromedial portal. Under direct arthroscopic visualization, the area of the detached ligament was penetrated. One or two sutures were used to penetrate the tibiotalar fascicle depending on the area of the detached ligament. By folding the suture thread in half, a loop and two ends were obtained. Once the clamp was removed, the loop and the suture ends were in the same (anteromedial) portal. One of the suture ends was introduced into the loop; by pulling the ends, the loop entered the joint and was locked while the ligament was grasped by the suture (Figure 3).

**Figure 4:**
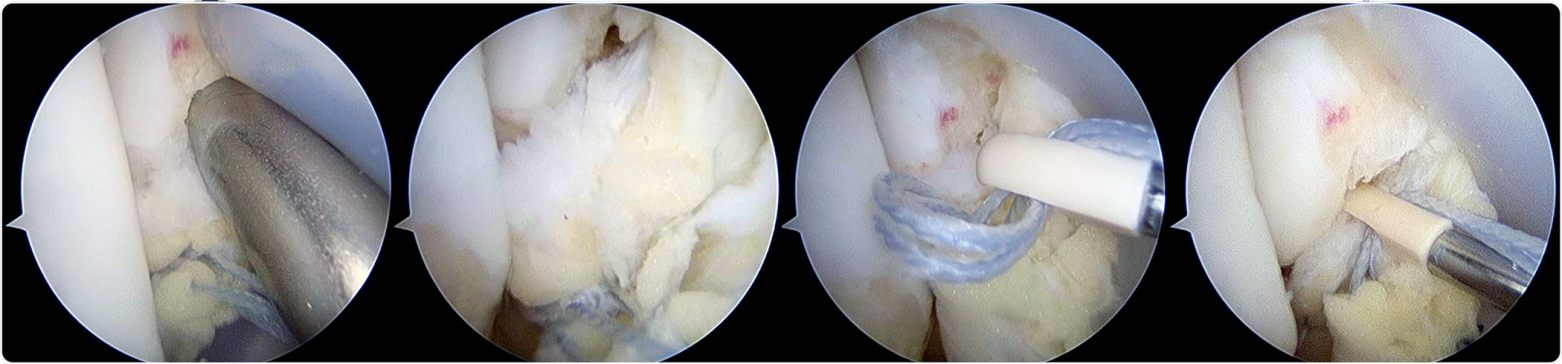
Arthroscopic view of a right ankle showing the drill guide placed through the anteromedial portal and centred on medial malleolus. After bone tunnel is performed, the anchor with the suture is introduced by impaction.

**Figure 5:**
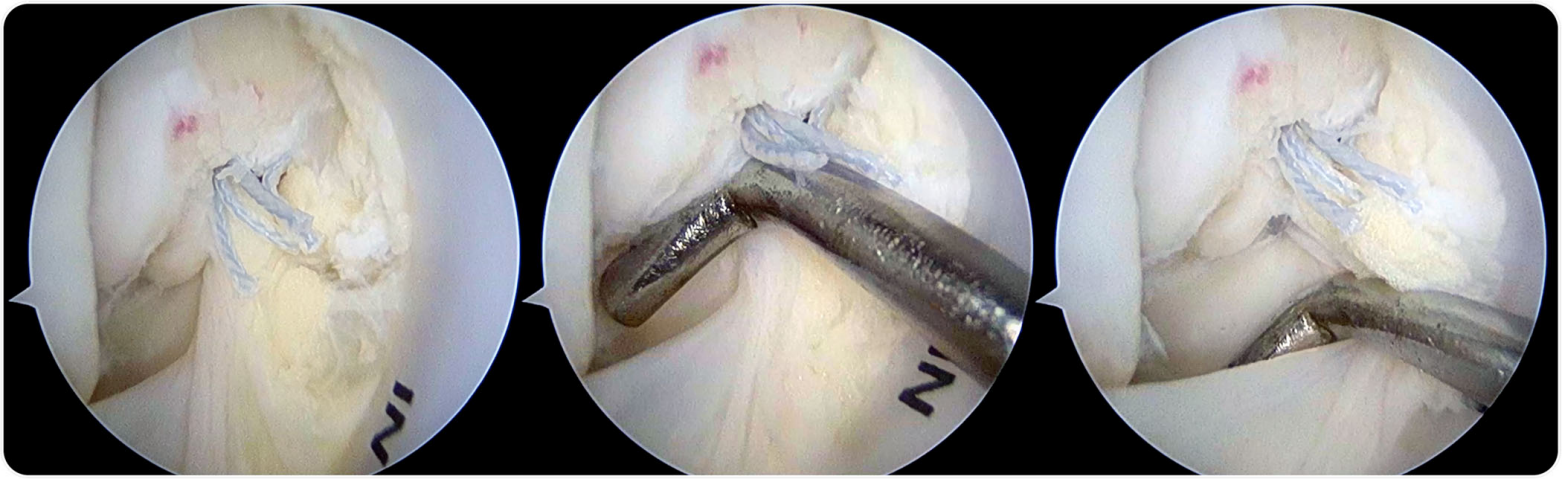
Arthroscopic view of a deltoid ligament injury after an all‐arthroscopic repair.

The appropriate location for the suture anchor was then identified. Optimal placement ought to be close to the tibiotalar fascicle detached area and on the anteromedial aspect of the medial malleolus. The anchor should be aimed at mid‐distance between the medial malleolus tip and the previously described location for the treatment of rotational ankle instability [29]. The drill guide was placed through the anteromedial portal and centred over the anchor's desired location. The drill had to be directed parallel to the plantar surface when the ankle is held at 90° and from anterior to posterior aiming medially at approximately 10°–15° to avoid penetration into the tibiotalar joint space. Once the hole was drilled, the bone anchor with the sutures was introduced by impaction **(**Figure 4). Tensioning of the suture was adjusted before the introduction of the anchor. The tibiotalar fascicle was reattached with the ankle positioned in dorsiflexion without rotation (Figure 5).

In the case of concomitant anterior MCL open book injury, this was arthroscopically repaired as previously described [29]. Next, the lateral collateral ligament was repaired as required using an arthroscopic all‐inside technique [30].

Venous thromboprophylaxis was given for 10–15 days following surgery. A removable walking boot was worn at all times for the first 3–4 weeks, and partial weight bearing was started after 2–3 weeks. Physical therapy was initiated 1 week after the surgery. The walking boot was removed for physical therapy sessions. The first stages of physical therapy entailed active and passive range of motion. Three weeks after surgery, at the second physiotherapy stage, gait training, strengthening in dorsiflexion, plantarflexion, eversion and inversion, as well as proprioceptive training with weight bearing, were introduced.

## RESULTS

Seven patients underwent an arthroscopic all‐inside medial and lateral ligament repair with a knotless suture anchor technique and were all retrospectively reviewed. They were followed up for a median time of 34 (range: 13–75) months, with no patients lost to follow‐up during the study period.

A tear affecting the tibiotalar fascicle at the level of the medial malleolus tip was observed in all cases. In two patients, the fibres of the tibiotalar fascicle were grossly preserved at its proximal insertion, resulting in a 'wave sign' injury from an arthroscopic view. In the rest of the patients, a complete separation was observed between the ligament and the medial malleolus. An anterior MCL open book injury was also observed in one of the patients, indicating rotational ankle instability added to the posttraumatic MAI. Arthroscopic examination of the lateral collateral ligaments revealed five patients with an isolated detachment of the ATFL and two patients with both the ATFL and calcaneofibular ligament (CFL) involved.

All patients underwent additional arthroscopic ankle procedures. These included chondral talar lesion debridement in one patient, osteophyte resection in five, and hindfoot endoscopy in three. In those undergoing hindfoot endoscopy, an os trigonum excision and flexor hallucis longus release were performed in all three patients, alongside the removal of intra‐articular posteromedial loose bodies in one.

At the final follow‐up, all patients reported subjective improvement in their ankle instability. The feeling of collapse of the medial arch disappeared in all cases. On clinical examination, the anterior drawer test was negative and no medial or lateral talar tilt was observed in any patient. All patients returned to their daily activities without difficulties and to their preinjury level of sports performance without limitation. No deficit in ankle range of motion was observed when compared to the contralateral side.

No complications were encountered. Four patients reported pain or complaints along the course of the posterior tibialis tendon during the rehabilitation process. This was relieved with the use of plantar orthosis providing medial arch support.

The median AOFAS score increased from 68 (range: 64–70) preoperatively to 100 (range: 90–100) at final follow‐up.

## DISCUSSION

The most important contribution of this study is the description of the first arthroscopic all‐inside anatomic repair of the tibiotalar fascicle after a traumatic event and the presentation of the first series of patients who underwent successful surgical treatment.

As an all‐arthroscopic procedure, advantages of surgical treatment in posttraumatic MAI are those of less surgical aggression and faster recovery. The arthroscopic findings in this patients' series should help expand the knowledge of MAI after a traumatic event. Depending on the location of the MCL injury observed during arthroscopy, and according to its insertion on the anterior colliculus, the pathology can be divided into those affecting the ligaments attached to the anterior aspect of the anterior colliculus (anterior or precollicular MCL fascicle) or those affecting the ligament attached to the tip of the anterior colliculus (intermediate or collicular MCL fascicle). The description of the intermediate or collicular MCL fascicle injury presented in this series, which was consistently observed, could be considered to be pathognomonic of posttraumatic and dynamic MAI. A high index of suspicion is paramount in recognizing this injury when performing an arthroscopic ligamentous repair.

Although the MCL has been widely described in anatomy books, only limited reports exist that describe the MCL from an arthroscopic point of view [14, 29]. The MCL is a strong and multifascicular ligament that spans from the medial malleolus to the navicular, talus and calcaneus. Anatomic descriptions are variable, and most investigators agree that the MCL is composed of two fascicles, superficial and deep [2, 3, 4, 8, 9, 15, 23, 24].

**Figure 6:**
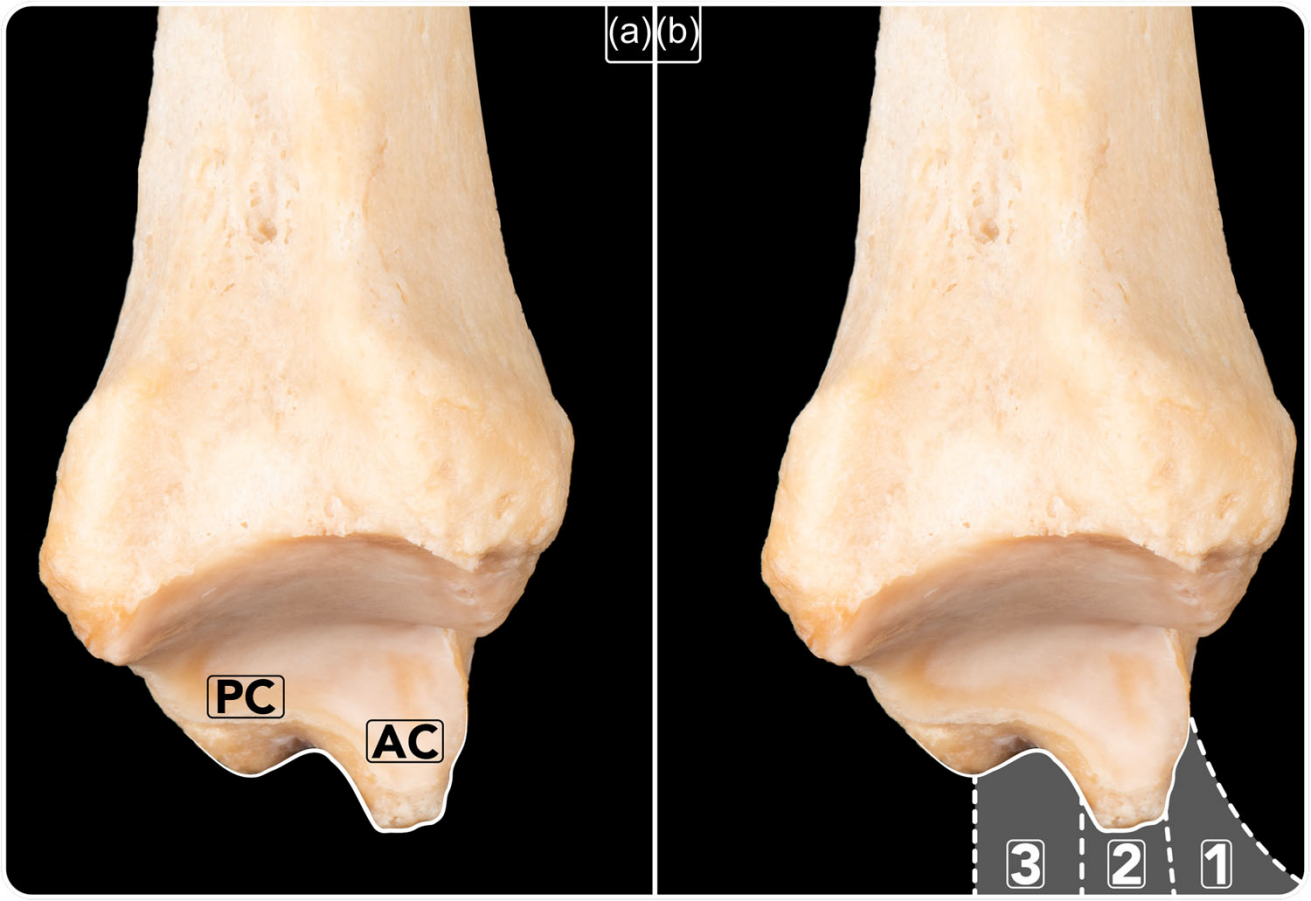
Bony anatomy of the lateral (intra‐articular) part of the tibial malleolus. (a) AC: Anterior colliculus; PC: posterior colliculus. (b) The three parts of the tibiotalar fascicle of the deltoid ligament have been outlined: 1, Anterior or precollicular (anterior part of the anterior colliculus). 2, Intermediate or collicular (anterior colliculus). 3, Posterior or postcollicular (posterior to the anterior colliculus).

From an arthroscopic point of view, only the deep MCL fascicle (tibiotalar fascicle) can be observed. The arthroscopic vision of the medial gutter with the arthroscope introduced through the anterior portals consists of the medial malleolus, the medial wall of the talus and the deep MCL fascicle. The deep MCL fascicle is arthroscopically observed as a hammock from the medial malleolus to the medial wall of the talus. This is usually observed as a single layer that does not allow individual fascicles to be differentiated. The medial malleolus in its anterior vision is usually observed with a thin fringe of cartilage that articulates with the medial wall of the talus, followed by a central fringe of cortical bone and the insertion of the deep MCL fascicle. The tip of the medial malleolus, when observed arthroscopically, corresponds to its anterior colliculus. With the anterior colliculus as a reference and based on the ligament's attachment to the anterior colliculus, the deep MCL can be arthroscopically divided into three components: anterior or precollicular, intermediate or collicular and posterior or postcollicular (Figure 6).

The anterior deep MCL component is attached to the anterior border of the anterior colliculus. Injuries affecting the precollicular component are seen in cases of rotational ankle instability when concomitant ATFL injuries are present [29]. MCL open book injury affecting the anterior fascicles is only observed when talar internal rotation or when instruments push away the ligament medially. In these cases, the MCL anterior fascicle is observed separating from the medial malleolus and giving the appearance of an open book. Recently, MCL open‐book injuries have been graded into two types depending on their degree of severity [31].

The deep MCL intermediate or collicular component is one that attaches on the anterior colliculus. As described here, injuries to deep MCL intermediate component were observed in patients after posttraumatic MAI. Although the MCL can probably be injured throughout its length, in the current study, the ligament injury has always been found arthroscopically detached off the medial malleolus at its proximal attachment and never at the mid‐portion or distal attachment. An injury to the deep MCL collicular component can be partial or complete as observed in the study.

The deep MCL posterior or postcollicular component is attached at the level of the intercollicular notch and the posterior colliculus. It is not fully visible when performing an anterior ankle arthroscopy under no‐distraction and dorsiflexion technique.

These anatomic features are crucial for correct recognition of a posttraumatic MCL injury. Any arthroscopic vision that differs from the usually described on the medial gutter should raise the suspicion of an MCL tear.

MAI is defined as a feeling of giving way localized medially when walking or during sports, and typically an asymmetric hindfoot valgus can be observed during weight‐bearing examination. MAI may be secondary to a number of causes. In the current study, all patients presented with concomitant lateral ligament complex deficiency from previous ankle injuries. Under weight‐bearing conditions, ATFL‐deficient ankles demonstrate increased anterior translation, internal rotation and superior translation of the talus [5]. As previously reported, patients with lateral ankle instability are more susceptible to injuries to the MCL and to develop MAI or rotational ankle instability as a consequence [14, 27]. From the published literature, the incidence of posttraumatic MAI in patients without ankle fractures has not been clearly reported. In our study, it was observed in only seven patients from 529 patients requiring surgery with a diagnosis of ankle instability, and it would represent an incidence of 1.3% among all ankle instability surgical candidates. In all cases, an injured lateral collateral ligament complex was also observed. It is uncertain whether there is an association between the two injuries, but from the described mechanisms of injury, it appears unlikely as the lateral structures are injured during inversion forces, whereas the medial ones are injured during eversion.

In all cases, the observed injury to the deep MCL was at the level of the anterior colliculus tip. The intermediate or collicular component of the deep MCL is proximally detached from the medial malleolus while its distal attachment remains intact. The injury arthroscopically observed corresponded to the deeper MCL (tibiotalar fascicle). However, it is difficult to ascertain if the superficial layer was injured, either partially or fully, as it is not observed arthroscopically. Imaging studies can help to determine the extent of MCL injury.

The MCL prevents eversion of the ankle joint and limits anterior, posterior and lateral displacement of the talus [6, 10, 11, 12, 15, 16]. The tibiotalar fascicle as the deep layer of the MCL is considered the major contributor to ankle stability against valgus forces, while the superficial layer contributes to stability to a lesser extent [17, 22, 25]. In the current study, the shortest and deepest fibres of the MCL were injured in all patients. As observed, and from a clinical point of view, patients reported MAI or medial foot arch collapse feeling after intense or stressful activity. However, when explored, no evident hindfoot pronation was observed, and symptoms were reproduced after running on the treadmill for more than 5 min. It is our hypothesis that patients with a partial MCL injury are susceptible to posterior tibialis muscle overuse. Consequently, muscular fatigue appears rapidly, and it is then when pronation and medial arch collapse feeling manifest, constituting an alert sign for dynamic MAI diagnosis.

The arthroscopic all‐inside repair of the collicular component of the deep MCL, with ligament fibres attached to the tip of the anterior colliculus, showed excellent results with a median AOFAS score that increased from 68 (range: 64–70) preoperatively to 100 (range: 90–100) at final follow‐up. No medial giving way or hindfoot valgus was observed during clinical examination at the final follow‐up, and no feeling of MAI and/or collapse feeling of the medial arch was reported during sports activity.

Study limitations include the lack of a comparative control group that underwent an open MCL repair. The benefits of treating injuries arthroscopically include the well‐known advantages of a minimally invasive approach. Through the same arthroscopic portals, all associated injuries as well as both the medial and lateral collateral ligaments can be treated. In addition, direct visualization and repair of injured ligament fibres are possible. Being the shorter ligament fibres the first to be injured in MAI, to access the injured deep layer of the MCL through an open procedure, a section of the superficial layer is required, while they will never be sectioned when arthroscopically addressed. On the other side, because concomitant pathologies were treated in the current study, it is not possible to assert the real effect of the isolated deltoid ligament arthroscopic repair. Finally, the AOFAS score used is not a validated outcome scale nor a specific item to evaluate ankle instability, and consequently, some clinical aspects may have been overlooked. A specific clinical score to assess ankle instability would have increased the validity of the study.

The clinical relevance of the study is that the intermediate or collicular deep MCL fascicle injury should be considered pathognomonic of posttraumatic and dynamic MAI.

## CONCLUSION

This study has shown that posttraumatic MAI can be successfully treated arthroscopically. The presence of an MCL tear affecting the deep and intermediate or collicular component of the ligament or tibiotalar fibres attached to the area of the anterior colliculus tip should be considered a sign of posttraumatic MAI. This partial deltoid injury at the level of the intermediate or collicular component will conduct to a dynamic MAI.

## AUTHOR CONTRIBUTIONS

**Jordi Vega**: Conceptualization; methodology; writing. **Francesc Malagelada**: Methodology; writing; editing. **Matteo Guelfi**: Methodology; writing; editing. **Miki Dalmau‐Pastor**: Methodology; writing; editing.

## CONFLICT OF INTEREST STATEMENT

The authors declare no conflict of interest.

## ETHICS STATEMENT

This study was approved by the ethical committee of Quirón Hospital (ID number of the approval: 10‐01‐2020). A written informed consent was obtained from all the patients.

## Data Availability

The authors confirm that the data supporting the findings of the study are available within the article.
